# *In Vivo* Noninvasive Imaging of Healthy Lower Lip Mucosa: A Correlation Study between High-Definition Optical Coherence Tomography, Reflectance Confocal Microscopy, and Histology

**DOI:** 10.1155/2013/205256

**Published:** 2013-09-03

**Authors:** Alejandra García-Hernández, Rodrigo Roldán-Marín, Pablo Iglesias-Garcia, Josep Malvehy

**Affiliations:** Melanoma Unit, Dermatology Department, Hospital Clínic, C/Villaroel 170, 08036 Barcelona, Spain

## Abstract

In recent years, technology has allowed the development of new diagnostic techniques which allow real-time, *in vivo*, noninvasive evaluation of morphological changes in tissue. This study compares and correlates the images and findings obtained by high-definition optical coherence tomography (HD-OCT) and reflectance confocal microscopy (RCM) with histology in normal healthy oral mucosa. The healthy lip mucosa of ten adult volunteers was imaged with HD-OCT and RCM. Each volunteer was systematically evaluated by RCM starting in the uppermost part of the epithelium down to the lamina propia. Afterwards, volunteers were examined with a commercially available full-field HD-OCT system using both the “slice” and the “en-face” mode. A “punch” biopsy of the lower lip mucosa was obtained and prepared for conventional histology. The architectural overview offered by “slice” mode HD-OCT correlates with histologic findings at low magnification. In the superficial uppermost layers of the epithelium, RCM imaging provided greater cellular detail than histology. As we deepened into the suprabasal layers, the findings are in accordance with physiological cellular differentiation and correlate with the images obtained from conventional histology. The combined use of these two novel non-invasive imaging techniques provides morphological imaging with sufficient resolution and penetration depth, resulting in quasihistological images.

## 1. Introduction

In recent years, technology has allowed the development of new diagnostic techniques which allow real-time, *in vivo*, noninvasive acquisition of images to evaluate morphological changes in tissue. These techniques include high-frequency ultrasound, reflectance confocal microscopy (RCM), and optical coherence tomography (OCT). The use of these technologies has proven to have application in ophthalmology [1], cardiology [2], gastroenterology [3], dermatology [4–6], and vascular surgery [7]. 

Oral malignancy is particularly high among men and the 8th most common cancer worldwide [8]. It represents about 2% of cancers in the UK [9] and 3% of all cancers in men and 2% of all cancers in women within the United States [10]. Squamous cell carcinoma accounts for about 90% of cases of oral cancer [11]. The majority of them develop from premalignant lesions, which generally appear as white patches (leukoplakia) or red patches (erythroplakia). Their malignant transformation rates are reported to be 0.9% to 17% and 14% to 50%, respectively [12]. The difficulty of determining whether such lesions are merely dysplasias or have already progressed to invasive carcinomas represents a constant therapeutic dilemma. Additionally, tumor detection is further complicated by the tendency toward field cancerization leading to multicentric lesions [13]. In the last 2 decades, diagnostic progress has been limited and overall patient survival has remained more or less unchanged. Unfortunately, advances in surgery, radiotherapy, medical oncology, and chemotherapy have offered limited progress in terms of overall survival. Until today, the single greatest determinant of long-term patient survival and quality of life remains early lesion detection and prompt and effective treatment [10]. 

Apart from excisional biopsy, there does not exist other validated methods for detecting malignant transformation *in vivo*. In such a manner, a sensitive diagnostic tool for noninvasive detection of oral malignancy and reliable screening of high-risk populations would be very helpful to identify patients at early, more treatable stages of the disease [14]. One of the main aims of these different, *in vivo*, noninvasive technologies is the identification of premalignant disorders and cancer at its earliest stage. 

Optical coherence tomography (OCT) is a relatively new biomedical imaging modality that can generate high-resolution cross-sectional images of microstructures in biological systems, resembling vertical sections in histology [1]. Only recently has OCT been used more systematically for the investigation of oral cavity lesions [10, 15–17]. High-definition optical coherence tomography (HD-OCT) is a technique based on the principle of conventional OCT, specifically the ability to carry out optical imaging deeply within highly scattering media, with micrometre resolution in both transversal and axial directions. It uses a two-dimensional, infrared-sensitive (1000–1700 nm) imagining array for light detection. This enables focus tracking: the focal plane is continuously moved through the sample. The movements of the focal plane and the reference mirror are synchronized, and the refractive index of the sample is taken into account. This results in a high lateral resolution of 3 *μ*m at all depths of the sample. A high axial resolution (3 *μ*m in skin) is achieved using a broadband thermal light source combined with a special filter. Moreover, the system is capable of capturing a slice image and an en-face image in real time as well as fast (35 s) 3D acquisition. The spectral sensitivity makes it possible to work in the near infrared range above 1000 nm. The field of view is 1.8 × 1.5 mm. The tissue penetration depth goes up to 570 *μ*m, and the total light power at the tissue is <3.5 mW. The system works in direct contact with the tissue using an optical matching gel comparable to ultrasound gel. The result is transferred to a computer and displayed using a greyscale or color palette, thereby generating an HD-OCT image. 

Reflectance confocal microscopy (RCM) is an noninvasive imaging technique that enables an en-face (horizontal plane) visualization of the tissue with a resolution at the cellular level (0.5–1.0 *μ*m in the lateral dimension and 4-5 *μ*m in the axial ones) and therefore may provide an alternative to histopathology. The device uses a diode laser as a source of monochromatic and coherent light, which penetrates into the tissue and illuminates a small point inside. The light is reflected, goes through a small pinhole and forms an image in the detector. This small pinhole does not allow the reflected light (reflectance) to reach the detector from another point. Therefore, only reflected light from the focal region (confocal) is detected. Light from out-of-focus planes is rejected at the pinhole. The contrast in RCM images relies on the differences in the reflectivity of the tissue, which is depending on chemical and molecular structures. Due to these variations of the refractive index, only a certain portion of the light is reflected. Structures with a higher refractive index appear bright in RCM. Melanin and melanosomes are the strongest natural sources of contrast resulting white in RCM. The commercially used RCM Vivascope 1500 uses a laser diode with a near-infrared wavelength of 830 nm. Laser power of 830 nm causes no damage to tissue, but limits the imaging depth to 200–300 *μ*m. The correlation between images of RCM and histopathology in oral mucosa has been previously reported [18, 19]. 

This study compares and correlates the images obtained by HD-OCT and RCM with histology findings in normal healthy oral lower lip mucosa. Thus, it serves as a foundation for the use of these noninvasive imaging techniques as screening tools for the early detection of oral cancer and precancer. 

## 2. Materials and Methods

### 2.1. Subjects

The healthy oral lower lip mucosa of ten adult volunteers was imaged with HD-OCT and RCM. All gave their informed consent. The study population consisted of 7 women and 3 men ranging in age between 22 and 56 years old. Four subjects were skin phototype III, four were phototype IV, and two were phototype II. Demographic data of study population is summarized in Table 1.

### 2.2. Evaluation Protocol

**Figure 1:**
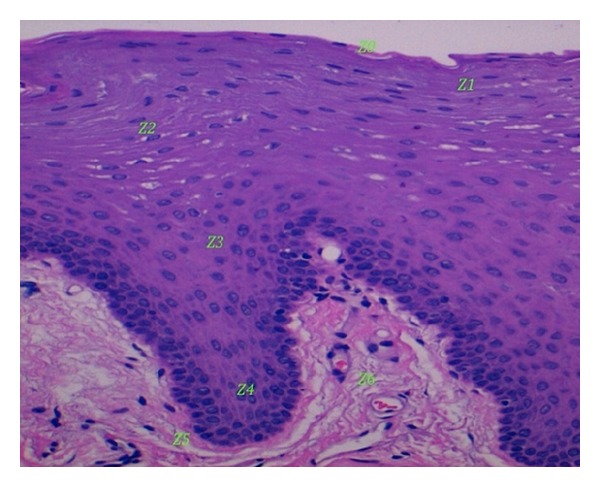
Histology image of healthy oral lower lip mucosa with reference marks (Z) showing where standardized depth reflectance confocal microscopy evaluation was peformed.

A commercially available Reflectance Confocal Microscope (Vivascope 1500 Caliber I.D., New York, USA) was used for imaging. A detailed description of this technique and the device used has been previously published. Reflectance confocal microscopy evaluation was performed on the oral mucosa of the lower lip. Each volunteer was systematically evaluated by RCM: 6 individual images were obtained at previously set standardized depths with the reference mark (Z0) set in the uppermost part of the epithelium down to the lamina propia (Figure 1), and vertical mapping using a Vivastack was performed in 1.5 *μ*m steps to a maximum depth of 150 *μ*m beginning at the uppermost part of the epithelium into the lamina propia. 

After the evaluation with RCM, all volunteers were examined with a commercially available full-field HD-OCT system (Skintell, Agfa Healthcare Morstel, Belgium and München, Germany). A detailed description of the technique and the device has been previously published. All volunteers were systematically evaluated. As a result of the large diameter of the HD-OCT probe, there was no possible identification mark within the mucosa. The probe was placed in the centre of the lower lip oral mucosa to allow a rough colocalization with the RCM and histological section. To make an image, the subject was positioned in front of the system. The probe was lifted from the holder, and optical gel (approximately 10 *μ*L) was applied to the probe sensor lens. The probe sensor lens was then positioned on the patient's oral mucosa using mild pressure. Scanning automatically began in the slice mode. A live “slice” view of the mucosal tissue was displayed, the image was captured and saved. During scanning, the foot pedal was used to switch between slice mode and en-face mode. A 3D scan was was also performed. Characteristic features were documented.

### 2.3. Histological Evaluation

Two volunteers underwent a 3 mm “punch” biopsy of the lower lip mucosa following local anesthesia with 2% lidocaine with epinephrine, and tissue sections were prepared as usual for conventional histology with H&E. The histological evaluation was performed by a board-certified pathologist and a dentist specialized in oral medicine and oral pathology (AGH).

## 3. Results

### 3.1. Epithelial Uppermost Layers

**Figure 2:**
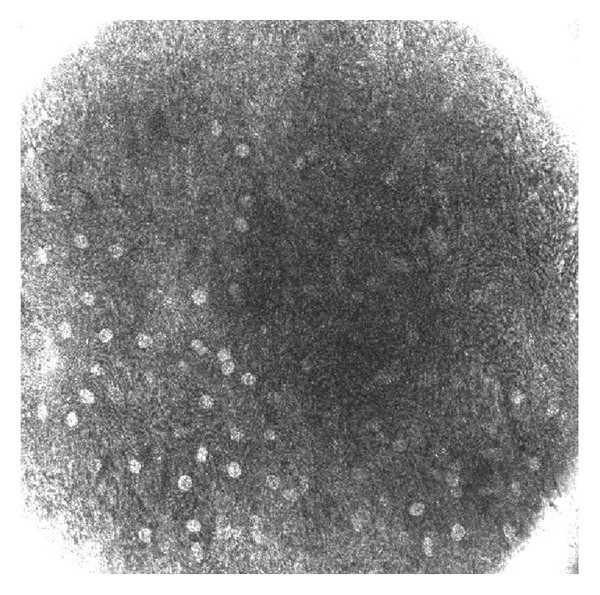
Reflectance confocal microscopy image at Z0 showing ill-demarcated cells with bright nuclei surrounded by a dark halo.

With RCM in the superficial uppermost layers of the epithelium (Z0), we visualized a highly scattering image where we observed mild scaling, and cells appeared flattened and ill-demarcated. Nuclei appeared bright and were surrounded by a dark halo (Figure 2). 

**Figure 3:**
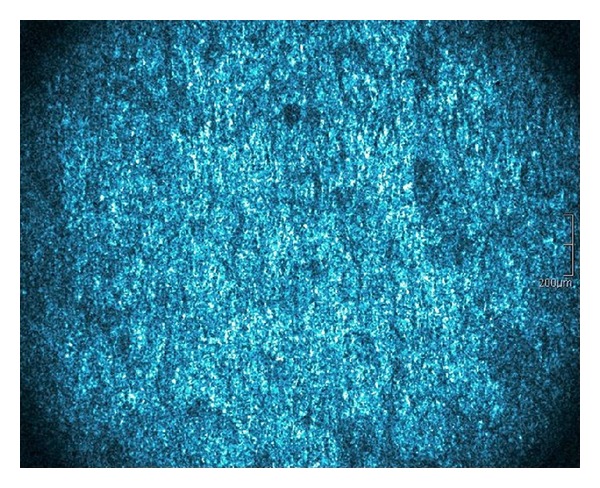
High-definition optical coherence tomography “en-face” image at supreficial epithelial layers.

The en-face mode of HD-OCT starting in the uppermost part of the epithelium demonstrated a highly scattering image where architectural epithelial structure could be appreciated, but horizontal imaging with HD-OCT did not achieve cellular detail resolution as RCM (Figure 3). 

**Figure 4:**
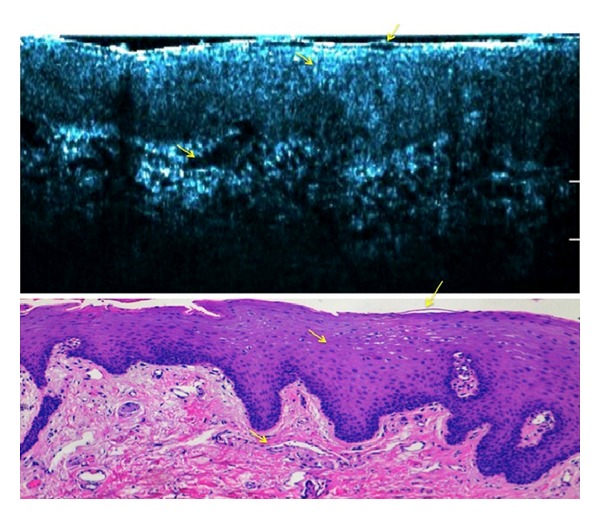
High-definition optical coherence tomography “slice” image with histologic correlation. Yellow arrows pinpoint the anatomical correlation between the “slice” image obtained with HD-OCT and the histology image.

When imaging the oral mucosa with HD-OCT in real time “slice” view, the reflectivity of the tissue led to a signal-intense, bright band on the surface, which corresponds with the entrance signal. The thinly, partially parakeratinized layer appeared as a scattering homogenous layer, with a sharp demarcation line between it and the higher scattering epithelium. The architectural overview offered by “slice” mode HD-OCT correlates with histologic findings at low magnification (Figure 4).

**Figure 5:**
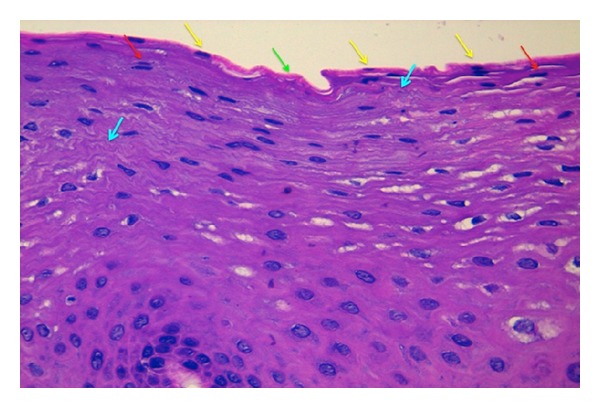
Conventional histology image under light microscopy showing the uppermost epithelial layers. Green arrow: non-keratinized area pinpointing abscence of nuclei. Yellow arrows: parakeratinized areas with presence of nuclei in the uppermost epithelial layer. Red arrows: flat nuclei in upper epithelial layers. Blue arrows: large filament content of cytoplasm giving appearance of microfolds.

Under the light microscope on conventional histology, the epithelium seemed as nonhomogenously parakeratinized. The cells in the uppermost layers of the epithelium appeared very eosinophilic, thin, and flat. Nuclei persisted and also became notoriously flattened. The large filament content of the cytoplasm may give the appearance of microfolds. The intercellular space between the epithelial cells is narrow (Figure 5). 

### 3.2. Stratum Granulosum/Prickle Cell Layers

**Figure 6:**
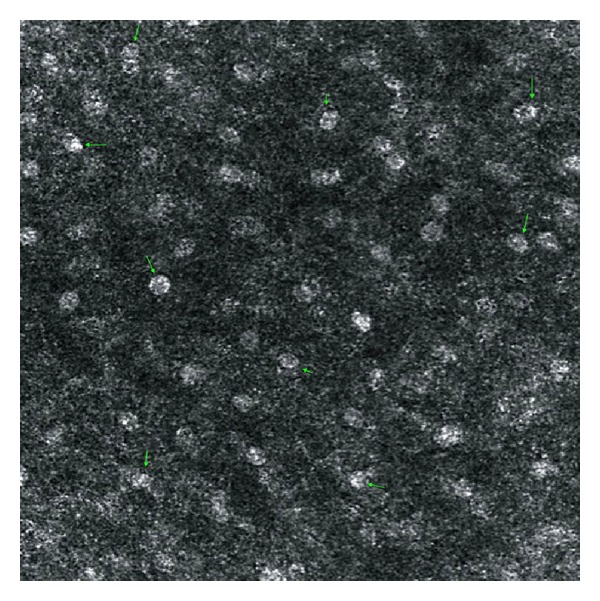
Reflectance confocal microscopy image at the stratum granulosum layer (Z1). Green arrows: nuclei display mostly as bright rounded structures and a fine granular apperance of the cytoplasm is observed.

**Figure 7:**
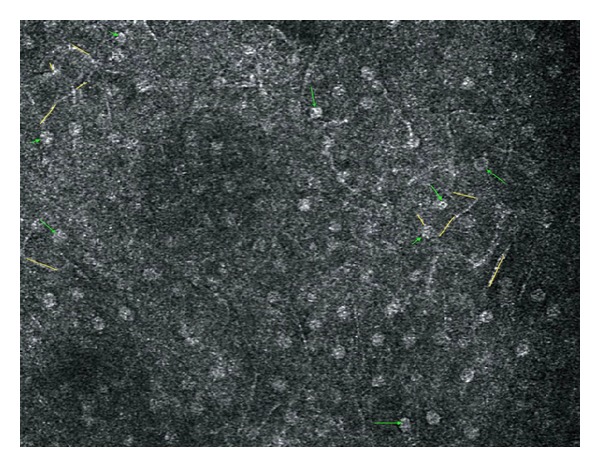
Reflectance confocal microscopy image at the prickle cell layer (Z2). Green arrows: signal bright rounded nuclei. Yellow lines: cytoplasmic border and intercellular junction.

**Figure 8:**
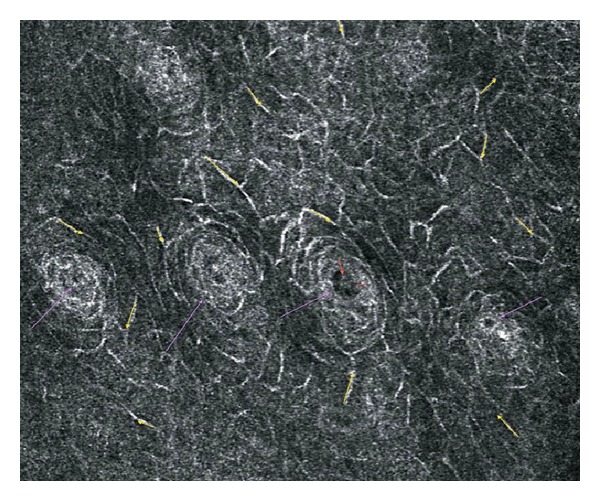
Reflectance confocal microscopy image at prickle cell layer (Z3). Yellow lines: reticular or wider honeycomb pattern, Purple arrows: dermal papillae, Red arrows: blood vessels.

With RCM at the stratum granulosum cells appeared to have a granular aspect possibly related to the presence of cytoplasmic granules (Z1). Nuclei are easily distinguished as bright mostly round structures (Figure 6). As we deepened into the epithelium to the suprabasal layers (Z2 and Z3), cells appeared larger, more spherical, and hyporefractive. Nuclei occupied a greater proportion of the total area of the cell and were clearly visible as bright structures. Cell membranes start becoming visible, and intercellular borders and junctions (desmosomes) become apparent (Figure 7). At Z3 level, the epithelium acquires a more reticular or widened honeycomb pattern. Dermal papillae may start becoming visible (Figure 8). These findings are in accordance with physiological cellular differentiation and correlate with the image obtained from conventional histology.

When visualizing these layers with the “en-face” mode of HD-OCT, cells appeared small and also revealed a granular appearance at the stratum granulosum layer correlating properly with the RCM and histology findings. In the stratum spinosum cells were clustered giving the appearance of a honeycomb pattern.

The “slice” mode of HD-OCT showed that the epithelium reveals a less intense signal than the lamina propia. 

On conventional histology, the granular layer is wanting. However, the transitional status of the cells is clearly recognized. Cells and nuclei become flat and irregularly shaped. Some nuclei acquire a dotted or pyknotic appearance. In the prickle cell layer, cells appear larger, round, or polygonal. Nuclei seem large and intensely basophilic and remain centrally located. 

### 3.3. Basal Cell Layer and Epithelial Junction

**Figure 9:**
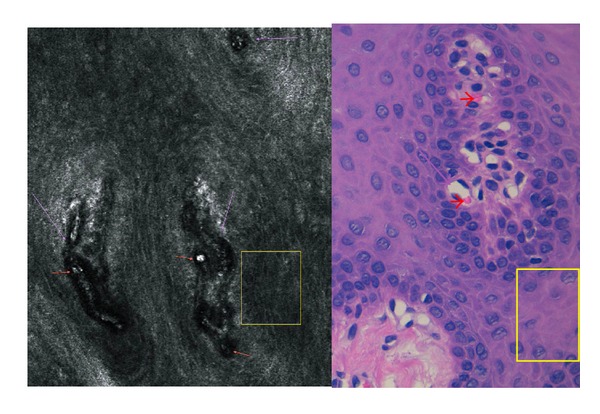
Reflectance confocal microscopy findings at the basal cell layer and epithelial junction with histologic correlation. Purple arrows: blood vessels, Red arrows: erythrocytes and blood vessel traffic, Yellow square: loss of honeycomb pattern.

With RCM, the basal cell layer (Z4 and Z5) at the interpapillary pegs cells appeared much smaller, condensed, and surrounding the papillae. In this plane, nuclei were visible as dark structures within cells. Within the papillae, small bright cells corresponding to erythrocytes traveling inside the blood vessels were easily distinguished (Figure 9).

HD-OCT “en-face” mode at the epithelial junction provided the architectural appearance of rings of bright cells surrounding the dark dermal papillae. With the “slice” mode, the epithelial junction could be distinguished if the border was relatively flat and not overly serrated.

On conventional histology, at the basal cell layer, cells have a cuboidal shape containing large rounded nuclei. Mitotic activity can be easily distinguished.

### 3.4. Lamina Propia

**Figure 10:**
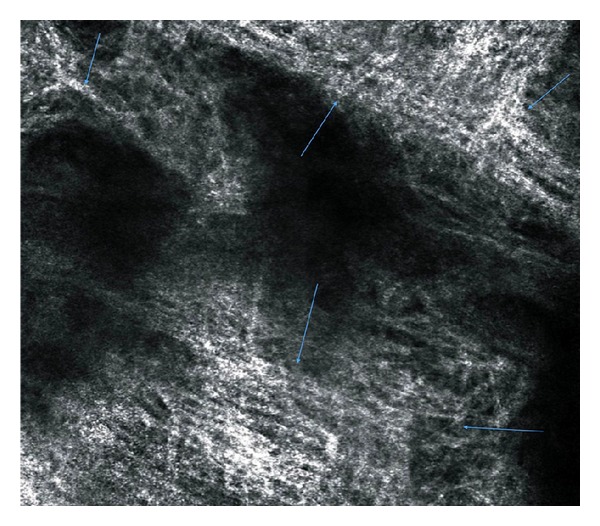
Reflectance confocal microscopy image at the lamina propia showing apperance of collagen fibers and bundles. Blue arrows: collagen fibers.

With RCM at the lamina propia (Z6) collagen fibers and blood vessels with active blood flow were visible, showing proper correlation with histological sections (Figure 10). 

**Figure 11:**
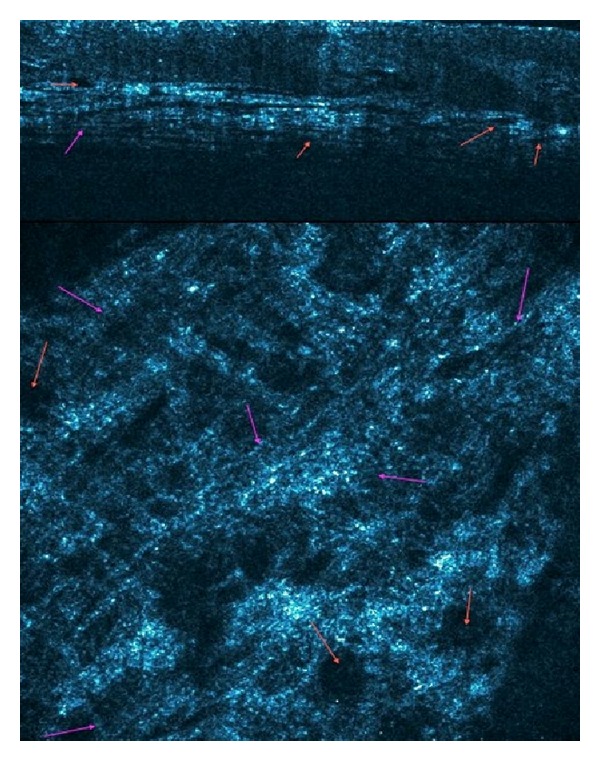
High-Definition Optical Coherence Tomography “slice” view and “en-face" image correlating findings at the level of the lamina propia. Purple arrows: collagen fibers, Red arrows: blood vessels.

In the lamina propia, “en-face” HD-OCT showed the refractive nature of collagen which makes it appear as white reticulated fibers with scattered blood vessels. It is worthwhile mentioning that the depth of penetration with which an image with fair-enough resolution could be observed with HD-OCT was approximately 450 *μ*m. While imaging with HD-OCT in “slice” mode the lamina propia appeared more signal-intense, with some signal poor cavities corresponding to blood vessels (Figure 11). 

On conventional histology, the lamina propia is composed of loose connective tissue with a rich vascular supply. The deeper layers contain heavy bundles of collagen fibers.

## 4. Discussion

The diagnosis of oral cancer and precancer still requires taking a surgical biopsy to study the sampled tissue histologically. Oral biopsies can be painful and associated with bleeding and difficulties in eating and speaking. Alternatively, different noninvasive optical diagnostic techniques have emerged. 

Reflectance confocal microscopy has revolutionized the noninvasive technique for morphological investigation of living tissue on a microscopic level. Unlike routine histology, the images obtained with RCM are parallel to the surface (horizontal or en-face), rather than cross-sectional (vertical or slice). They are greyscale images, similar to radiographic images. The field of view with reflectance confocal microscopy is 500-500 *μ*m across. The level being imaged can be identified by the morphological appearance of the tissue at a given depth. On the other hand, the depth of the section can be measured using a micrometre attached to the Z stage of the objective lens. With this system it is possible to image normal tissue to a depth of 200 *μ*m. The boundaries of individual cells are easily visualized because of the high lateral resolution of approximately 1 *μ*m and the axial resolution (section thickness) of 3–5 *μ*m. Reflectance confocal microscopy findings of healthy oral mucosa had been previously documented. However, these findings until now had not been validated. Our results confirm the validity of RCM imaging as an appropriate noninvasive tool to assess tissue architecture and cellular morphology of the oral cavity. According to our results in the vast majority of cases we were unable to achieve imaging with appropriate resolution beyond 195 *μ*m, and at this penetration depth our findings were consistent with structures found in the lamina propia. In contrast, White et al. [18] report lamina propia findings at a penetration depth of 350–450 *μ*m. It is possible that these disagreements in depth are related to the variability of epithelial thickness among subjects but also possibly related to differences in the illumination source. They used an Nd:YAG laser confocal microscope at 1064 nm, while we used a diode laser at 830 nm, thus penetration discordance may be expected. 

Just very recently, Contaldo et al. have published RCM features in different healthy oral mucosal sites (lips, cheek, gingivae, and tongue) and provided further evidence on the usefulness and applicability of the handheld version of RCM (Vivascope 3000, Lucid, Rochester, NY) as an adjunct tool in the clinical evaluation and management of patients with oral diseases [19]. 

Despite the significant progress in the *in vivo* analysis, RCM does have limitations. Firstly, images are only parallel to the tissue surface, and the penetration depth is limited. Moreover, RCM image interpretation requires a fair amount of specialized training; results remain strongly user dependent and reproducibility of results may vary between investigators. Furthermore, unfortunately RCM equipment is expensive and currently only available in few selected centres. Yet, interest in the field of confocal microscopy is growing and its usefulness is becoming increasingly evident. 

OCT has been recently used to assess oral cavity lesions and to distinguish benign from malignant lesions. However, these studies have been performed with SS-OCT and *ex vivo* tissue samples. To the best of our knowledge, this is the first study using HD-OCT in the *in vivo* examination of healthy oral mucosa.  Table 2 summarizes technical data differences between RCM, SS-OCT, and HD-OCT.

In contrast to RCM, images obtained with HD-OCT are both horizontal (en-face) and vertical (slice). The images generated are displayed using either greyscale or a color palette. The field of view of the en-face mode (parallel to the surface) is 1.8–1.5 mm across. A lateral resolution of 3 *μ*m allows the identification of individual cells. The high bandwidth of the lightsource leads to a high axial (depth) resolution of 3 *μ*m, which is comparable to that of RCM. With this system it is possible to visualize tissue to a depth of up to 570 *μ*m, which is almost three times deeper than with RCM. The main limitation for the use of HD-OCT to examine oral mucosal tissue is the size and weight of the probe which makes it impossible to access certain areas of the oral cavity. 

Another relevant and interesting finding from our results is that the lining mucosa of the lower lip revealed a thinly, nonhomogenously, parakeratinized epithelium. The majority of histology textbooks mention that the epithelium in this anatomical area is nonkeratinized. This may be due to a certain anatomical variability of this basic pattern in adults, as exemplified by the thinly keratinized linea alba (“white line”) at the level of the occlusal plane. 

The combined use of these two novel noninvasive imaging techniques provides morphological imaging with sufficient resolution and penetration depth (Tables 3, 4, and 5), resulting in quasihistological images. One can only imagine that in a near future the advances in noninvasive imaging techniques will allow *in vivo*, easy, fast, painless examination of inflammatory and proliferative diseases of the oral cavity.

In conclusion, our findings suggest that high-definition optical coherence tomography and reflectance confocal microscopy allow examination of healthy oral lower lip mucosa with adequate histological correlation. Further studies are needed to assess the validity of these avant-garde diagnostic techniques in diseased oral tissue. 

## Figures and Tables

**Table 1:** Demographic parameters of studied population.

Patients	Gender	Age	Phototype
1	F	29	III
2	M	37	IV
3	F	34	III
4	F	22	III
5	F	43	III
6	F	35	IV
7	F	56	II
8	M	31	IV
9	F	27	II
10	M	31	IV

F: Female; M: Male.

**Table 2:** Technical data on swept source-optical coherence tomography, high definition-optical coherence tomography, and reflectance confocal microscopy.

Technical data	SS-OCT Vivo Sight	HD-OCT Skintell	RCM VivaScope 1500
Light source	Santec HSL-2000-12-MDL	Halogen lamp with Gaussian filter	Diode laser
Wavelength	1310 nm	1300 nm	830 nm
Lateral resolution	<7.5 *μ*m	3 *μ*m	0.5 to 1.0 *μ*m
Axial resolution	<5 *μ*m	3 *μ*m	4 to 5 *μ*m
Penetration depth	1200 to 2000 *μ*m	570 *μ*m	200–300 *μ*m
Field of view (en-face)	6 × 6 mm	1.8 × 1.5 mm	0.5 × 0.5 mm 8 mm × 8 mm VivaStack
3D Imaging	Yes	Yes	No

nm: nanometre; *μ*m: micrometre; mm: millimetre.

**Table 3:** Reflectance confocal microscopy, high-definition optical coherence tomography, and conventional histology morphological characteristics in lip mucosa according to tissue depth.

Reference mark	Depth	RCM characteristics	HD-OCT “en-face” characteristics	Histology
Z0	0 *μ*m	Highly scattering homogenous	Highly scattering homogenous layer	Nonhomogenously parakeratinized
Z1	4.96 *μ*m	Cytoplasmic granular appearance	Granular appearance and high scattering	Wanting granular layer. Cells appear very eosinophilic, thin and flat. Narrow intercellular spaces
Z2	29.79 *μ*m	Cytoplasmic border becomes apparent	Cytoplasmic granular appearance and areas with honeycomb pattern	Cells and nuclei become flat and irregularly shaped. Nuclei acquire pyknotic appearance
Z3	84.36 *μ*m	Wider honeycomb pattern. May be difficult to clearly distinguish nuclei. Dermal papillae may start becoming visible	Dermal papillae may start becoming visible	In the prickle layer, cells appear round or polygonal. Nuclei seem intensely basophilic and centrally located
Z4	124.00 *μ*m	Loss of honeycomb pattern. Nuclei become dark and are not clearly distinguished	Dermal papillae	Basal cell layer cells have a cuboidal shape containing large rounded nuclei. Mitotic activity can be easily distinguished
Z5	173.00 *μ*m	Epithelial junction, ill-defined papillae and collagen fibers	Transitional epithelium	
Z6	193.69 *μ*m	Collagen fibers	Collagen fibers	Loose connective tissue with a rich vascular supply. The deeper layers contain heavy bundles of collagen fibers

**Table 4:** Cellular features in lower lip mucosa evaluated *in vivo* by reflectance confocal microscopy and high-definition optical coherence tomography.

Reference mark (*μ*m)	Cellular structure	RCM image from Vivastack sets (*μ*m)	HD-OCT image from “slice” mode (*μ*m)
Z0	Nuclei (where visible)	10–13.2	NA
Z1	Nuclei	12–14.3	9.7–13.5
Z2	Nuclei	11–16.8	12–17.6
Z3	Nuclei	10–14	10.9–16.9
Z4	Nuclei	NA	NA
Z5	Nuclei	NA	NA
Z6	Nuclei	NA	NA
Z0	Cytoplasmic border	21–34.2	24.6–29.4
Z1	Cytoplasmic border	22–27	21–22.9
Z2	Cytoplasmic border	23–35.4	22.4–35
Z3	Cytoplasmic border	25–34	21.9–33.8
Z4	Cytoplasm	21–32	18.8–20.2
Z5	Cytoplasm	16–22	NA
Z6	Cytoplasm	NA	NA
Z5-Z6	Junctional epithelium	16–32	20.5–29

NA: not applicable.

**Table 5:** Mean measurements of cellular and morphologic features in lower lip mucosa *in vivo * by reflectance confocal microscopy and high-definition optical coherence tomography.

Structure	Histology image (*μ*m)	RCM image (*μ*m)	HD-OCT image (*μ*m)
Epithelium thickness (*μ*m)	100–245	153–194	151–232
Interpapillary space	33–203.7	NA	57.4–498
Papillae diameter (shortest/longest) (*μ*m)	99/265	NA	150/287.1
Capillary diameter (longest/shortest) (*μ*m)	200/34	208/33	261.9/36.7
Collagen fiber bundles	Regularly dispossessed reticular pattern	Regularly dispossessed reticular pattern	Regularly dispossessed reticular pattern
